# Comparative plastome analyses and phylogenetic insights of *Blumea* DC

**DOI:** 10.3389/fpls.2026.1835658

**Published:** 2026-05-07

**Authors:** Pingxuan Xie, Junli Xie, Changliu Shao, Kailang Mu, Zhigang Ju, Xiaomin Tang, Yunheng Ji, Hanjing Yan, Yuan Yuan, Yuxin Pang

**Affiliations:** 1 Scool of Pharmacy, Guizhou University of Traditional Chinese Medicine, Guizhou, China; 2 Scool of Traditional Chinese Medicine, Guangdong Pharmaceutical University., Guangzhou, China; 3 CAS Key Laboratory for Plant Diversity and Biogeography of East Asia, Kunming Institute of Botany, Chinese Academy of Sciences, Kunming, Yunnan, China

**Keywords:** blumea, comparative analyses, nrDNA sequence, phylogeny, plastome

## Abstract

**Background:**

*Blumea* DC. comprises approximately 100 species with significant morphological diversity. Yet its plastome has rarely been systematically investigated. Previous studies had divided the genus into three main clades: the *B. lacera* clade, the *B. densiflora* clade, and the *B. balsamifera* clade; however, the phylogenetic positions of these clades remain ambiguous. Furthermore, the monophyly of *B. formosana*, *B. sinuata*, *B. megacephala*, *B. aromatica*, *B. axillaris*, and *B. hieraciifolia* is unresolved and requires further investigation. The exact phylogenetic position of *Cyathocline* Cass., which has been subsumed into *Blumea*, also remains unclear.

**Methods:**

To investigate plastome features in *Blumea*, plastomes from 23 species and two varieties (including 23 newly sequenced samples and two publicly available representatives) were compared. Comparative analyses assessed structural and sequence variation, divergence hotspots, simple sequence repeats (SSRs), and codon usage bias. Additionally, phylogenetic inference was performed using a dataset of 47 complete plastomes and 38 nrDNA sequences to reconstruct the backbone phylogeny and address the aforementioned phylogenetic problems.

**Results:**

All *Blumea* plastomes exhibited a typical quadripartite structure with sizes ranging from 150,779 bp to 151,281 bp and contained 113 unique genes (79 protein-coding genes, 30 tRNA genes, and four rRNA genes). IRscope and Mauve analyses revealed minimal structural variation among plastomes. Despite the genes flanking each junction being identical, the junction between LSC, SSC, and IRs regions was classified into four types based on the variations in the distances between genes and their respective junctions. Nine divergent hotspot regions were identified as candidate DNA barcodes for future species identification. Between 79 and 95 SSRs were detected per plastome, predominantly in the large single-copy region and mainly comprising mononucleotide repeats. The genus displayed mono-, di-, tri-, tetra-, penta-, and hexa-nucleotide repeats, with specific types and quantities varying among species. Codon usage bias analysis indicated conservation in preferred codon types, numbers, and RSCU values. Phylogenetic analyses consistently supported the division of *Blumea* into four clades: the *B. lacera* clade, *B. balsamifera* clade, *B. densiflora* clade, and *B. stricta* clade. Besides, the monophyly of *B. formosana*, *B. sinuata*, *B. aromatica*, and *B. hieraciifolia* was well supported.

**Conclusions:**

This study conducts the first large-scale comparative analysis of *Blumea* plastomes to date, systematically revealing the conservation and specificity of plastome features of the genus. Phylogeny based on plastome and nrDNA dataset has provided an enhanced phylogenetic framework, and preliminary clarified the phylogeny of the genus. Moreover, the monophyly of taxa including *B. formosana*, *B. sinuata*, *B. megacephala*, *B. aromatica*, *B. axillaris*, and *B. hieraciifolia* was well examined. In summary, this study provided substantial informative genetic data pertinent to *Blumea* and offered new insights into the phylogeny of *Blumea*, laying the foundation for subsequent taxonomic, systematic, and identification studies.

## 1 Introduction

*Blumea* DC. (Asteraceae) comprises approximately 100 species, primarily distributed in tropical Asia, with a few in Australia and Africa (Anderberg and Eldenäs, 2007). The genus includes annual or perennial subshrubs, shrubs, and herbs, and many taxa (e.g., *B. balsamifera* (L.) DC., *B. riparia* DC., *B. aromatica* DC., etc.) are of great medicinal value (Editorial Committee of Chinese Materia Medica of the National Administration of Traditional Chinese Medicine, 1999).

Due to its morphological diversity, the classification of *Blumea* has been controversial. Randeria (1960) proposed a widely adopted classification system, dividing the genus into seven sections (*Semivestitae*, *Macrophyllae*, *Sagittatae*, *Hieraciifoliae*, *Paniculatae*, *Oxyodontae*, and *Dissitiflorae*) based morphological characteristics. However, molecular studies have failed to support this arrangement, instead, grouping *Blumea* into three main clades: the *B. lacera* clade, *B. densifora* clade, and *B. balsamifera* clade (Pornpongrungrueng et al., 2007; Pornpongrungrueng et al., 2009; Chung et al., 2022).,Besides, these phylogenetic analyses based on single or multiple DNA loci (including *ITS*, *psbA*-*trnH*, and *trnL*-*trnF*) have produced inconsistent evolutionary trees, making the relationships among these clades unclear. Moreover, poor resolution and statistical support in these studies have left phylogenetic relationships within *Blumea* unresolved. Previous research has also failed to confirm the monophyly of *B. formosana* Kitam., *B. sinuata* (Lour.) Merr., *B. megacephala* (Randeria) C. C. Chang & Y. Q. Tseng, *B. aromatica*, *B. lacera* (N. L. Burman) DC., *B. axillaris* (Lam.) DC., *B. saxatilis* Zoll. & Moritzi, and *B. hieraciifolia* (Spreng.) DC (Pornpongrungrueng et al., 2007; Pornpongrungrueng et al., 2009; Chung et al., 2022). The genus *Cyathocline* Cass. was incorporated into *Blumea* based on molecular and karyotypic data (Li et al., 2014), with *C. purpurea* (Buch.-Ham. ex D. Don) Kuntze described as the synonym of *B. stricta* (DC.) Anderb. & Bengtson (Rajbhandari et al., 2024). Yet, the exact phylogenetic position of *Cyathocline* within *Blumea* is still unresolved. Therefore, to address these issues, a more comprehensive phylogenetic analysis with extensive sampling and genomic data is necessary.

To date, plastid genome (plastome) and nuclear ribosomal DNA (nrDNA) derived from genome skimming data are widely employed in phylogenetic studies, frequently exhibiting high analytical performance (Park et al., 2022; Xia et al., 2022; Fu et al., 2024). Plastomes of *Blumea* have also been reported, displaying a typical quadripartite structure comprising one large single-copy (LSC) region, one small single-copy (SSC) region, and two inverted repeat (IR) regions (Abdullah et al., 2021; Zhao et al., 2021; Cao et al., 2026). However, only a few plastomes of this genus have been deposited in GenBank, and systematic comparative or phylogenetic analyses based on plastomes remain lacking. Moreover, the number of nrDNA sequences of *Blumea* is also limited. In this study, we newly sequenced 35 accessions from 21 species and two varieties, as well as three outgroup species to obtain complete plastomes and nrDNA sequences. Combined with nine *Blumea* plastomes downloaded from GenBank, a final dataset comprising 47 complete plastomes and 38 nrDNA sequences was used. Our primary objectives are as follows: (1) investigate the plastome features of *Blumea*; (2) reconstruct an enhanced backbone phylogeny and preliminarily elucidate the phylogenetic relationships within the genus; and (3) examine the monophyly of *B. formosana*, *B. sinuata*, *B. megacephala*, *B. aromatica*, *B. axillaris*, and *B. hieraciifolia.*

## 2 Materials and methods

### 2.1 Plant sampling, DNA extraction, and sequencing

Fresh leaves from 38 samples were collected and dried with silica gel, 35 of which represent 21 species and two varieties of *Blumea*. The identifications of the samples were conducted by Professor Yuxing Pang, following the *Flora Reipublicae Popularis Sinicae* (FRPS) (Editorial Committee of the Flora of China, 1979), as well as relevant literature on new *Blumea* species (Li et al., 2025). Voucher specimens were deposited in the Herbarium of Guizhou University of Traditional Chinese Medicine, with collection details provided in **Supplementary Table 1**. Genomic DNA was extracted from 100 mg of silica gel-dried plant tissue using the CTAB method (Doyle and Doyle, 1987). Total DNA was fragmented into 150 bp for pair-end library preparation utilizing a NEBNext^®^ Ultra™ Illumina DNA Library Prep Kit (NEB, USA), in accordance with the manufacturer’s instructions. All genomic data were sequenced on the Illumina NovaSeq 6000 platform at Shenzhen Huitong Biotechnology Co., Ltd. (Shenzhen,China).

### 2.2 Assembling and annotation

After filtering the raw data using fastP v0.15.0 (Chen et al., 2018) with parameters -n 10 and -q 15, high-quality reads were obtained. Subsequently, these high-quality reads were employed to assemble plastome and nrDNA through assembled using GetOrganelle v1.7.7.1 (Jin et al., 2020) with the default settings, employing the plastome (MZ424304) and nrDNA (KF443294) sequences of *B. balsamifera* as reference and seed, respectively. The assembled plastomes were edited and corrected using Bandage v0.9.0 (Wick et al., 2015) then annotated using the CPGAVAS2 (Shi et al., 2019). Additionally, Geneious v10.2.3 (Kearse et al., 2012). was used to manually adjust the annotated plastome. Circular maps of the plastomes were generated using OrganellarGenomeDRAW (OGDRAW) (Greiner et al., 2019). The nr DNA sequences of *Blumea* were annotated in Geneious v10.2.3 (Kearse et al., 2012), with the nrDNA sequence of *B. balsamifera* (KF443294), *Cynara cardunculus* var. *scolymus* (L.) Fiori (XR_003067690, XR_003067462) as the references. GenBank accession numbers for the 38 newly generated plastomes and nrDNA sequences are listed in **Supplementary Table 1**. Combined with nine additional plastomes retrieved from GenBank (**Supplementary Table 2**), a total of 47 plastomes and 38 nrDNA sequences were used in follow-up analyses.

### 2.3 Genomic comparative analysis

Plastomes representing 23 species and two varieties were selected for subsequent comparative analyses (**Supplementary Table 3**). The junction regions of the LSC (large single copy region), IRs (inverted repeat regions), and SSC (small single copy region) among plastomes were compared and plotted using CPJSdraw (Li et al., 2023). After removing one copy of the IR region, the general structural characteristics of *Blumea* plastomes were further analyzed using Mauve v2.3.1 (Darling et al., 2004). Furthermore, the sequence divergence of the *Blumea* plastomes was assessed using mVISTA (Frazer et al., 2004) under the Shuffle-LAGAN alignment mode, with the plastome of *B. balsamifera* set as the reference. Nucleotide variability values (pi) for the aligned *Blumea* plastomes were calculated using DnaSP v5.0 (Librado and Rozas, 2009), with the window length and step size set to 400 bp and 200 bp, respectively. Coding and non-coding regions with pi value ≥ 0.018 were extracted using Geneious v10.2.3 (Kearse et al., 2012), and the number of variable sites and parsimony-informative (parsim-info) sites was assessed using MEGA X (Kumar et al., 2018).

Simple sequence repeats (SSRs) of *Blumea* plastomes were identified via MISA (http://pgrc.ipk-gatersleben.de/misa/). The minimum repeat thresholds were set to 10, 5, 4, 3, 3, and 3 for mono-, di-, tri-, tetra-, penta-, and hexa-nucleotide repeats, respectively. Additionally, the codon usage patterns of the core protein-coding genes (PCGs) in the plastomes were analyzed using the Sequence Manipulation Suite Version 2 (SMS v2; https://www.detaibio.com/sms2/index.html), and the corresponding relative synonymous codon usage (RSCU) values were calculated.

### 2.4 Phylogenetic analyses

To infer the phylogeny of *Blumea* species, phylogenetic trees were reconstructed based on plastomes and nrDNA with *Laggera crispata* (Vahl) Hepper & J. R. I. Wood, *Elephantopus scaber* L., and *Duhaldea cappa* (Buch.-Ham. ex DC.) Anderb. were selected as outgroups according to Zhang et al. (2019) and Li et al. (2014) (**Supplementary Tables 1**, **2**). All plastomes and nrDNA sequences were aligned using MAFFT v7.450 (Katoh and Standley, 2013) implemented in Geneious v10.2.3 (Kearse et al., 2012) with the default settings, and then manually adjusted. The characteristics of each alignment were analyzed using MEGA X (Kumar et al., 2018). Phylogenetic trees were reconstructed using both maximum likelihood (ML) and Bayesian inference (BI) methods, with the optimal nucleotide substitution model (GTR+I+G4) determined by ModelTest-NG v1.2.2 (Darriba et al., 2020). Maximum likelihood trees were then constructed with RAxML-NG (Kozlov et al., 2019), with 1000 bootstrap replicates. Bayesian inference phylogenetic trees were performed using MrBayes v3.2.7 (Ronquist et al., 2012) running 1,000,000 Markov chain Monte Carlo (MCMC) generations, with sampling performed every 100 generations and the first 25% of generations disregarded as burn-in for each matrix. Stationarity was attained when the average standard deviation of split frequencies was less than 0.01. Phylogenetic trees were visualized and edited using FigTree v1.4.2 (Rambaut, 2014).

## 3 Results

### 3.1 Recovery of complete *Blumea* plastomes

**Figure 1:**
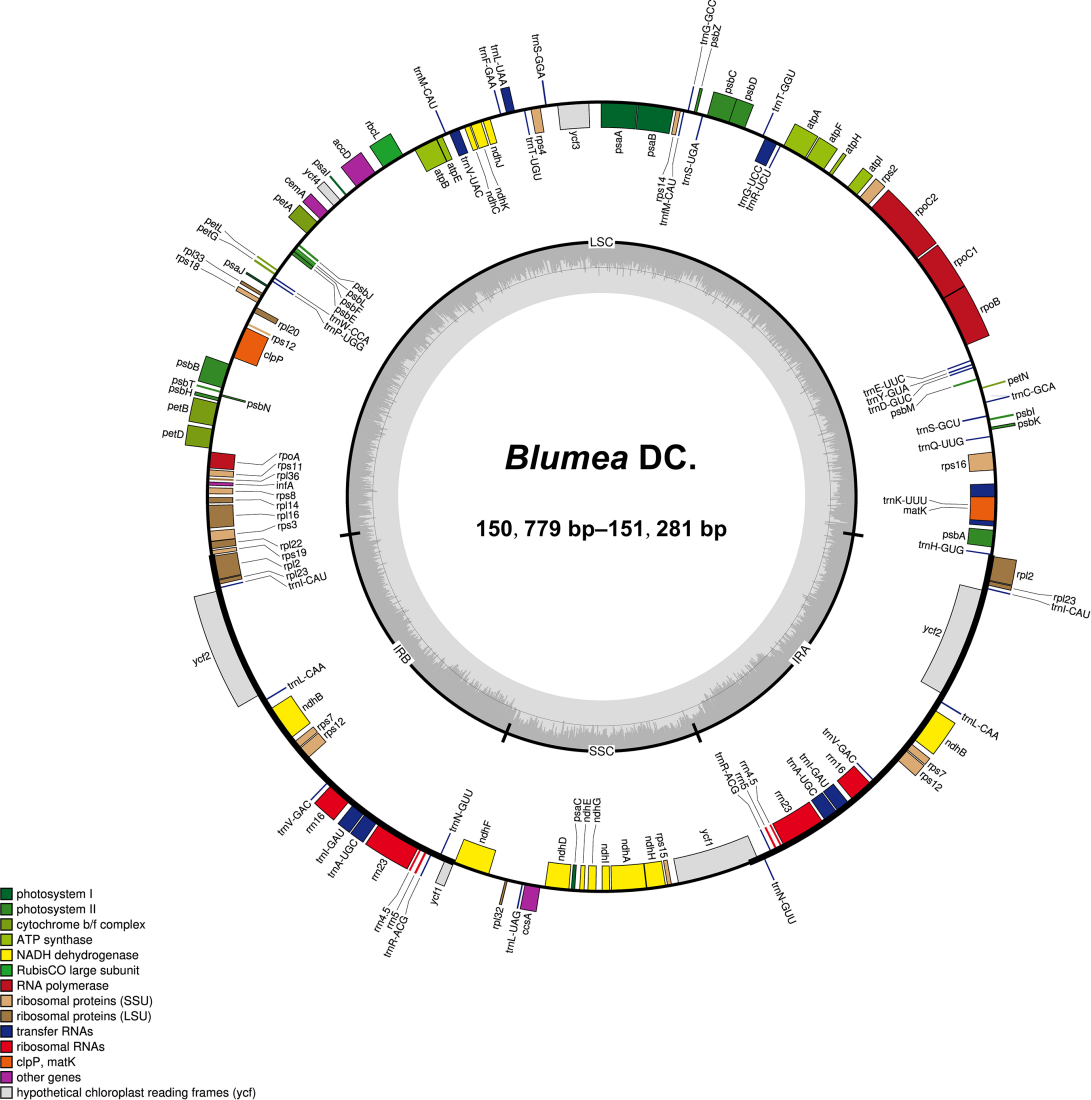
Circular maps of *Blumea* plastomes. The inside and outside of the circle are genes transcribed clockwise and counterclockwise, respectively. The genes belonging to different functional groups are color-coded. The dashed area in the inner circle shows GC content.

The summary of the low-coverage genome sequencing and assembly of plastomes was presented in **Supplementary Table 4**. The plastomes of *Blumea* ranged in length from 150,779 bp to 151,281 bp and exhibited a typical quadripartite structure (**Figure 1**; **Table 1**), consisting of one large single copy (LSC) region (82,478–82,897 bp), one small single copy (SSC) region (18,353–18,510 bp), and two inverted repeat (IR) regions (24,907–24,987 bp). The total GC content of the plastomes was 37.5%–37.6%. All *Blumea* plastomes encoded 113 unique genes, including 79 protein-coding genes, 30 transfer RNA (tRNA) genes, and 4 ribosomal RNA (rRNA) genes (**Table 1**; **Supplementary Table 5**). The features of nrDNA were listed in **Supplementary Table 6**.

**Table 1:** Comparison of plastome features among Blumea taxa in this study.

Samples/Accession number	Species	Size (bp)				Total GC content (%)	Gene number (unique)			
		Genome	LSC	SSC	IR		Total	PCGs	rRNA	tRNA
cp001	*B. balsamifera*	151,171	82,729	18,468	24,987	37.6%	113	79	4	30
cp002	*B. aromatica*	151,186	82,814	18,480	24,946	37.5%	113	79	4	30
cp003	*B. aromatica*	151,186	82,814	18,480	24,946	37.5%	113	79	4	30
cp007	*B. stricta*	151,087	82,668	18,499	24,960	37.5%	113	79	4	30
cp008	*B. martiniana*	151,251	82,878	18,501	24,936	37.5%	113	79	4	30
cp009	*B. lanceolaria*	151,170	82,819	18,477	24,937	37.5%	113	79	4	30
cp010	*B. sagittata*	151,144	82,786	18,464	24,947	37.5%	113	79	4	30
cp014	*B. sinuata*	150,788	82,530	18,354	24,952	37.6%	113	79	4	30
cp015	*B. sinuata*	150,790	82,532	18,354	24,952	37.6%	113	79	4	30
cp018	*B. aromatica*	151,186	82,814	18,480	24,946	37.5%	113	79	4	30
cp019	*B. henryi*	151,269	82,883	18,508	24,939	37.5%	113	79	4	30
cp020	*B. axillaris*	151,054	82,762	18,440	24,926	37.5%	113	79	4	30
cp022	*B. calcicola*	151,169	82,793	18,484	24,946	37.5%	113	79	4	30
cp024	*B. megacephala*	151,077	82,752	18,435	24,945	37.5%	113	79	4	30
cp026	*B. aromatica*	151,186	82,814	18,480	24,946	37.5%	113	79	4	30
cp028	*B. oblongifolia*	151,067	82,757	18,420	24,945	37.6%	113	79	4	30
cp029	*B. clarkei*	151,017	82,705	18,412	24,950	37.5%	113	79	4	30
cp030	*B. sinuata*	150,779	82,522	18,353	24,952	37.6%	113	79	4	30
cp034	*B. napifolia*	151,232	82,897	18,431	24,952	37.5%	113	79	4	30
cp038	*B. megacephala*	151,071	82,757	18,424	24,945	37.6%	113	79	4	30
cp039	*B. axillaris*	151,043	82,755	18,438	24,925	37.5%	113	79	4	30
cp041	*B. megacephala*	151,079	82,765	18,424	24,945	37.5%	113	79	4	30
cp044	*B. sessiliflora*	150,925	82,656	18,355	24,957	37.6%	113	79	4	30
cp047	*B. axillaris*	151,049	82,761	18,438	24,925	37.5%	113	79	4	30
cp051	*B. riparia*	151,046	82,740	18,418	24,944	37.6%	113	79	4	30
cp069	*B. eberhardtii*	151,118	82,789	18,417	24,956	37.5%	113	79	4	30
cp073	*B. hieraciifolia*	150,874	82,558	18,390	24,963	37.5%	113	79	4	30
cp074	*B. hieraciifolia*	150,871	82,557	18,390	24,962	37.5%	113	79	4	30
cp079	*B. fistulosa*	150,992	82,685	18,407	24,950	37.5%	113	79	4	30
cp104	*B. sericans*	151,211	82,876	18,431	24,952	37.5%	113	79	4	30
cp113	*B. hieraciifolia*	150,865	82,551	18,390	24,962	37.5%	113	79	4	30
cp118	*B. densiflora* var. *hookeri*	151,272	82,886	18,510	24,938	37.5%	113	79	4	30
cp122	*B. densiflora* var. *densiflora*	151,264	82,880	18,510	24,937	37.5%	113	79	4	30
cp127	*B. formosana*	151,260	82,885	18,503	24,936	37.5%	113	79	4	30
cp128	*B. formosana*	151,256	82,875	18,505	24,938	37.5%	113	79	4	30
PX394520	*B. axillaris*	151,043	82,755	18,438	24,925	37.5%	113	79	4	30
PX404823	*B. axillaris*	151,063	82,777	18,438	24,924	37.5%	113	79	4	30
NC_077558	*B. balsamifera*	151,170	82,740	18,466	24,982	37.6%	113	79	4	30
PX404818	*B. formosana*	151,281	82,894	18,509	24,939	37.5%	113	79	4	30
PX404820	*B. megacephala*	151,042	82,736	18,418	24,944	37.6%	113	79	4	30
PX404822	*B. sinuata*	150,783	82,524	18,355	24,952	37.6%	113	79	4	30
BK013127	*B. balsamifera*	151,176	82,746	18,466	24,982	37.6%	113	79	4	30
BK013128	*B. oxyodonta*	150,997	82,745	18,438	24,907	37.5%	113	79	4	30
BK013129	*B. tenella*	150,829	82,478	18,439	24,956	37.6%	113	79	4	30

### 3.2 Structural features across plastomes

**Figure 2:**
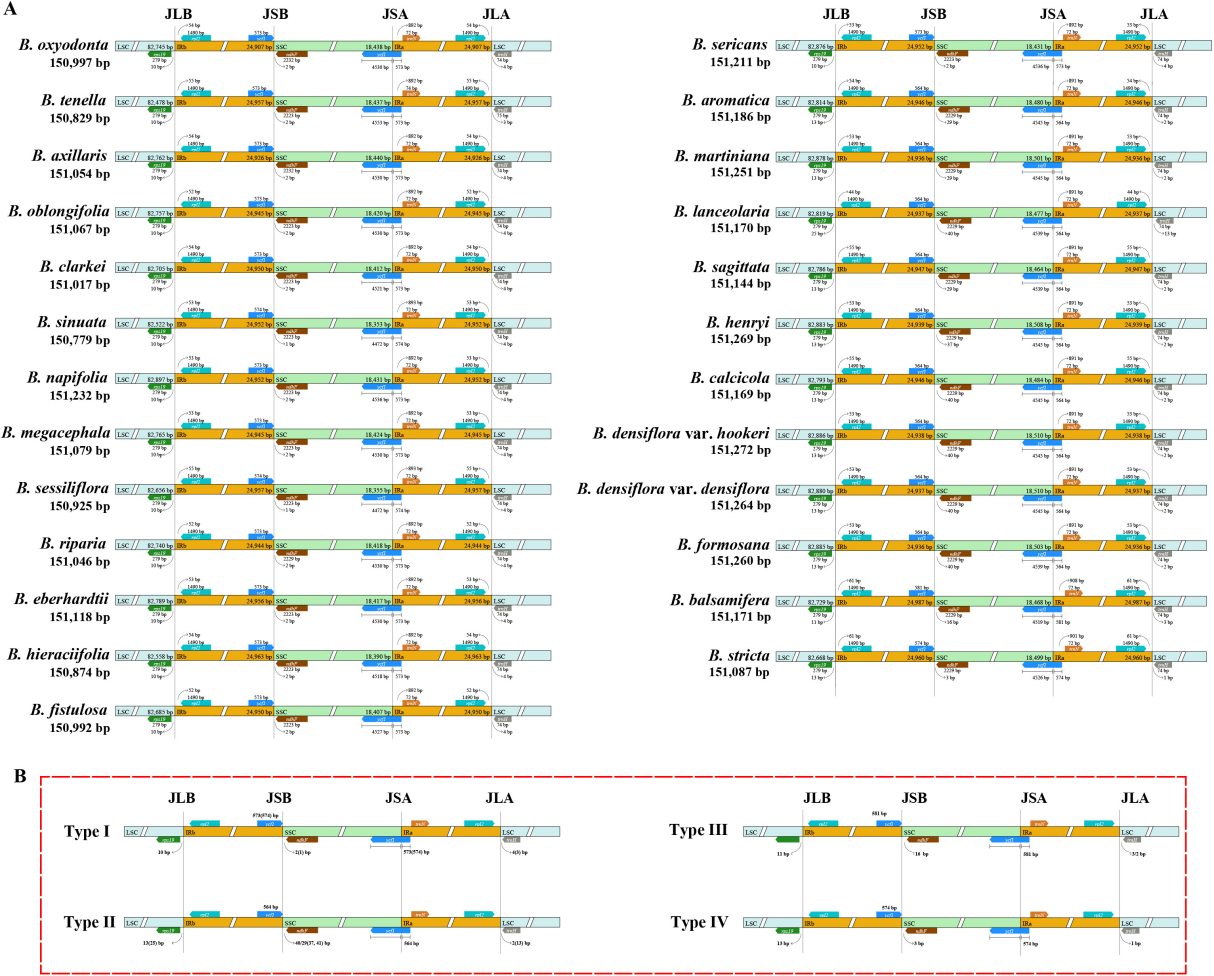
**(A)** Junctions of the LSC/SSC/IR regions of the *Blumea* plastomes; **(B)** Four types of junctions of the LSC/SSC/IR regions. JLB, junction line between LSC and IRb; JSB, junction line between IRb and SSC; JSA, junction line between SSC and IRa; JLA, junction line between IRa and LSC.

The junction between LSC and IRb (JLB) was located between the *rps19* and *rpl2* genes across all taxa; the junction between IRb and SSC (JSB) was downstream of the *ndhF* gene; the junction between SSC and IRa (JSA) was located within the *ycf1* gene; and the junction between IRa and LSC (JLA) lay between the *rpl2* and *trnH* genes (**Figure 2A**).

Furthermore, based on the distances of the *rps19*, *ndhF*, *ycf1*, and *trnH* genes to each junction (**Supplementary Table 7**), the junction patterns of *Blumea* plastomes could be broadly classified into four types (**Figure 2B**). Type I was characterized by a 10 bp distance between *rps19* and JLB, a two bp distance between *ndhF* and JSB (one bp in a few taxa), a 573 bp (574 bp in a few taxa) overlapping region between *ycf1* and IRs, and a four bp distance between *trnH* and JLA (three bp in a few taxa). This type mainly included *B. axillaris*, *B. clarkei* Hook. f., *B. eberhardtii* Gagnep., *B. fistulosa* (Roxb.) Kurz, *B. hieraciifolia*, *B. megacephala*, *B. napifolia* DC., *B. oblongifolia* Kitam., *B. oxyodonta* DC., *B. riparia*, *B. sericans* (Kurz) Hook. f., *B. sessiliflora* Decne., *B. sinuata*, and *B. tenella* DC.; Type II featured a 13 bp distance between *rps19* and JLB (25 bp in a few taxa), mostly 40 bp or 29 bp between *ndhF* and JSB (37 bp and 41 bp in a few taxa), a 564 bp *ycf1*-IR overlapping region, and a two bp distance between *trnH* and JLA (13 bp in a few taxa), and was observed in *B. aromatica*, *B. calcicola* XiongLi & K.W. Luo, *B. densiflora* var. *densiflora* DC., *B. densiflora* var. *Hookeri* (C. B. Clarke ex Hook. f.) C. C. Chang & Y. Q. Tseng, *B. formosana*, *B. henryi* Dunn, *B. lanceolaria* (Roxb.) Druce, *B. martiniana* Vaniot, and *B. sagittata* Gagnep.; Type III had an 11 bp distance between *rps19* and JLB, 16 bp between *ndhF* and JSB, a 581 bp *ycf1*-IR overlapping region, and a three bp distance between *trnH* and JLA (two bp in a few taxa). This type was restricted to the *B. balsamifera*; Type IV was only present in the *B. stricta*, with a 13 bp distance between *rps19* and JLB, a 3 bp distance between *ndhF* and JSB, a 574 bp *ycf1*-IR overlapping region, and a one bp distance between *trnH* and JLA. Mauve analysis revealed a highly conserved gene order in *Blumea* plastomes, with no gene inversion or rearrangement detected (**Supplementary Figure 1**).

### 3.3 Comparison of sequence variation

**Figure 3:**
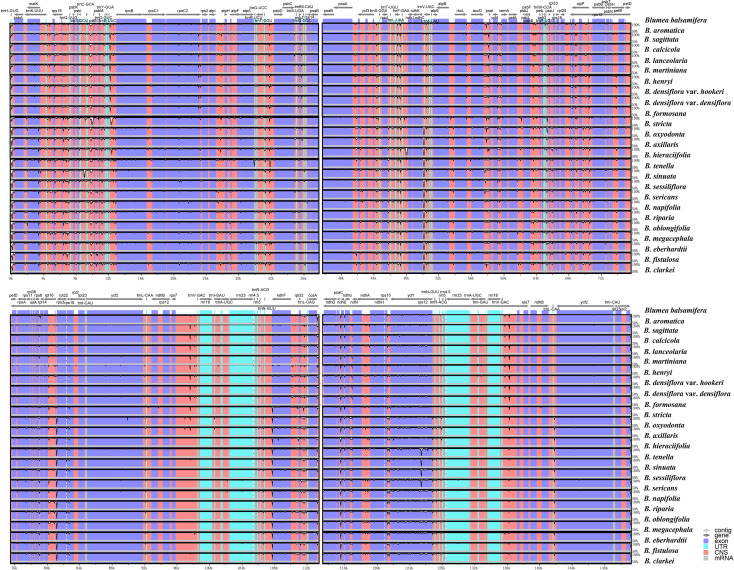
mVISTA visualization of the alignment for the 25 *Blumea* plastomes.

mVISTA analysis showed overall high sequence similarity across the genus (**Figure 3**). Sequence similarity was significantly lower in non-coding regions than in coding regions, most notably in intergenic spacers (IGSs). Among them, four coding regions (*matK*, *ndhF*, *ycf1*, and *rpoC2*) and 15 non-coding regions (*trnH*-*psbA*, *trnK*-*rps16*, *rps16*-*trnQ*, *trnS*-*trnC*, *trnC*-*petN*, *petN*-*psbM*, *trnY*-*rpoB*, ndhA intron, *trnT*-*psbD*, *accD*-*psaI*, *psbE*-*petL*, *ndhF*-*rpl32*, *rpl32*-*trnL*, *ccsA*-*ndhD*, and *rps15*-*ycf1*) displayed relatively high differences.

**Figure 4:**
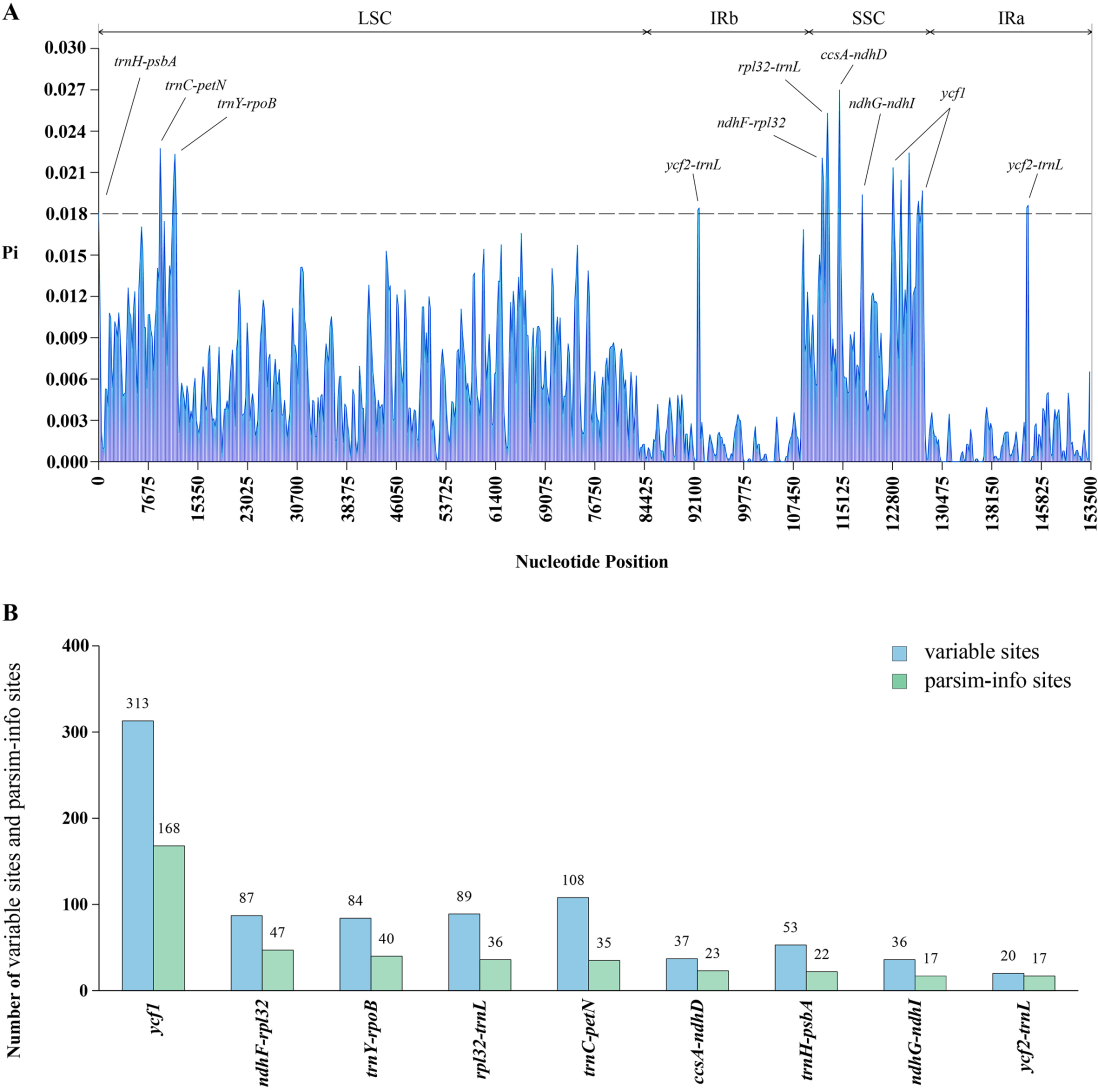
**(A)** Sliding window analysis of the 25 *Blumea* plastomes to show high variable regions. X-axis, position of the midpoint of a window; Y-axis, nucleotide variability values (p) of each window. The dashed line indicates that the p value is equal to 0.018; **(B)** Number of variable sites and parsim-info sites in the alignment of divergence hotspots.

The result sliding-window analysis revealed that the LSC and SSC regions harbored most high-Pi peaks, whereas the IRs region showed low nucleotide diversity (**Figure 4A**). Using a Pi threshold of 0.018, nine highly variable regions (*ycf1*, *trnH*-*psbA*, *trnC*-*petN*, *trnY*-*rpoB*, *ycf2*-*trnL*, *ndhF*-*rpl32*, *rpl32*-*trnL*, *ccsA*-*ndhD*, and *ndhG*-*ndhI*) were identified as divergence hotspots for *Blumea.* Among these, *ycf1* had the highest number of variable sites (313) and parsimony-informative sites (168). Although *trnC*-*petN* (108) and *rpl32*-*trnL* (89) ranked second and third in variable sites, their parsimony-informative sites (35, 36) were fewer than those of *ndhF-rpl32* (47) and *trnY-rpoB* (40) (**Figure 4B**).

### 3.4 Characterization of SSRs and codon usage bias

**Figure 5:**
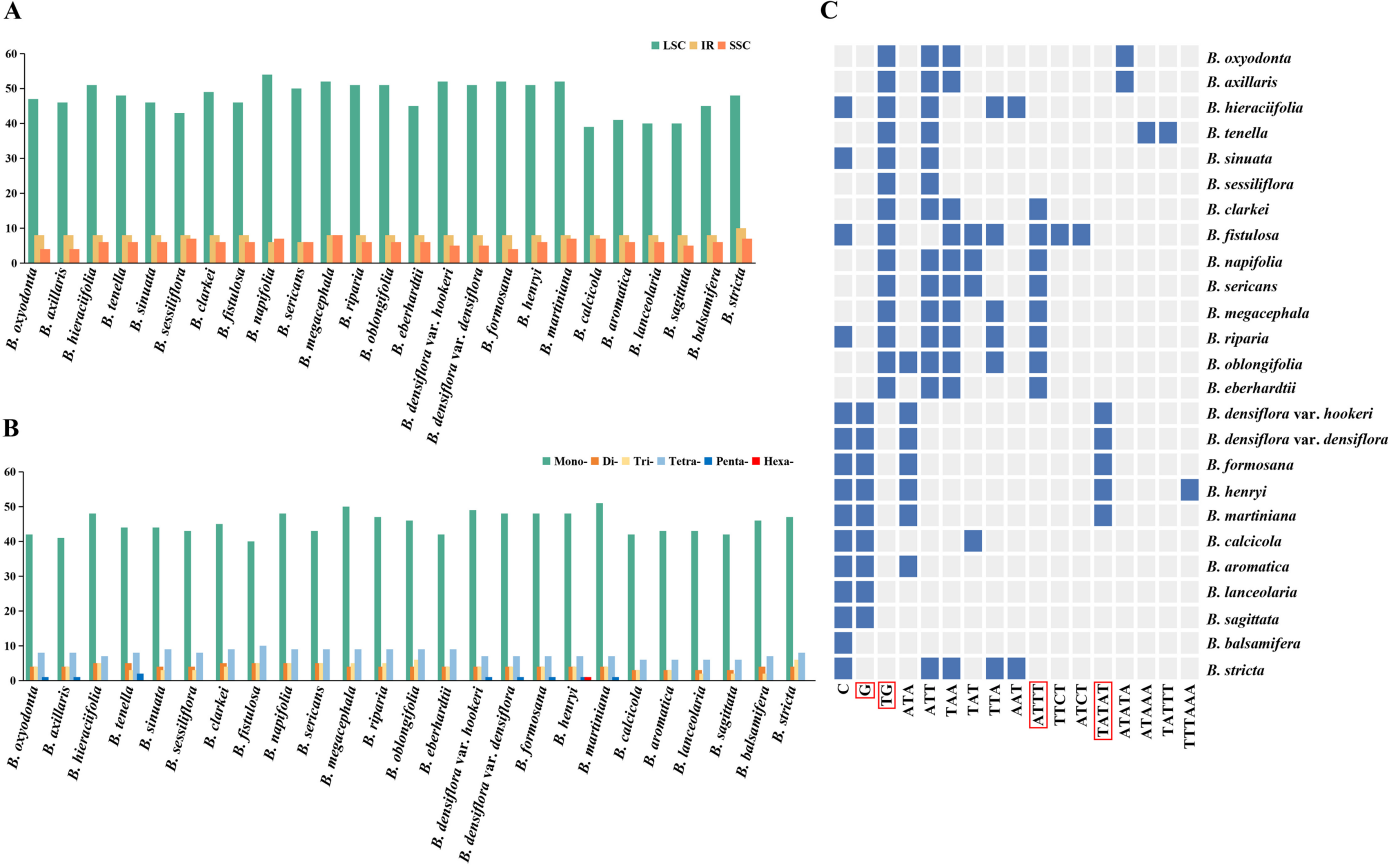
Analyses of simple sequence repeats (SSRs) in 25 *Blumea*s plastomes: **(A)** distribution of SSRs in LSC, IRS, and SSC regions; **(B)** number of different SSR types; **(C)** distribution of non-shared SSR motifs across different species.

A total of 53–68 simple sequence repeats (SSRs) were detected across the 25 *Blumea* plastomes (**Supplementary Table 8**). *B. megacephala* harbored the most SSRs (68), while *B. sagittata* had the fewest (53). These SSRs were predominantly distributed in the LSC region (39–54), with fewer in the SSC (4–8) and IR regions (6–10) (**Figure 5A**). All *Blumea* plastomes contained mono-, di-, tri-, and tetra-nucleotide repeats. Pentanucleotide repeats were only found in *B. axillaris*, *B. oxyodonta*, *B. tenella*, *B. henryi*, *B. martiniana*, *B. densiflora* var. *densiflora*, *B. densiflora* var. *hookeri*, and *B. formosana*, whereas hexanucleotide repeats were restricted to *B. henryi* (**Figure 5B**).

Among all SSRs, mononucleotide repeats were the most abundant (40–51), predominantly A/T motifs. Tetranucleotide repeats were the second most abundant (6–10), with dominant motifs AAAT, AATA, ATTG, TATT, TTAT, and TTTC. Dinucleotide (3–5) and trinucleotide (2–6) repeats were relatively rare (**Supplementary Table 8**; **Figures 5A, B**). Additionally, 17 non-shared SSR motifs were identified across taxa, and several motifs exhibited species-specific distributions (**Supplementary Table 8**; **Figure 5C**). Specifically, the G motif was detected in *B. densiflora* var. *hookeri*, *B. densiflora* var. *densiflora*, *B. formosana*, *B. henryi*, *B. martiniana*, *B. calcicola*, *B. aromatica*, *B. lanceolaria*, and *B. sagittata*; the TG motif occurred in *B. oxyodonta*, *B. axillaris*, *B. hieraciifolia*, *B. tenella*, *B. sinuata*, *B. sessiliflora*, *B. clarkei*, *B. fistulosa*, *B. napifolia*, *B. sericans*, *B. megacephala*, *B. riparia*, *B. oblongifolia*, and *B. eberhardtii*; the ATTT motif was found in *B. clarkei*, *B. fistulosa*, *B. napifolia*, *B. sericans*, *B. megacephala*, *B. riparia*, *B. oblongifolia*, and *B. eberhardtii*; and the TATAT motif was only present in *B. densiflora* var. *hookeri*, *B. densiflora* var. *densiflora*, *B. formosana*, *B. henryi*, and *B. martiniana*. Notably, some unique SSR motifs were taxon-specific, such as TTTAAA in *B. henryi*, ATAAA/TATTT in *B. tenella*, and TTCT/ATCT in *B. fistulosa*.

**Figure 6:**
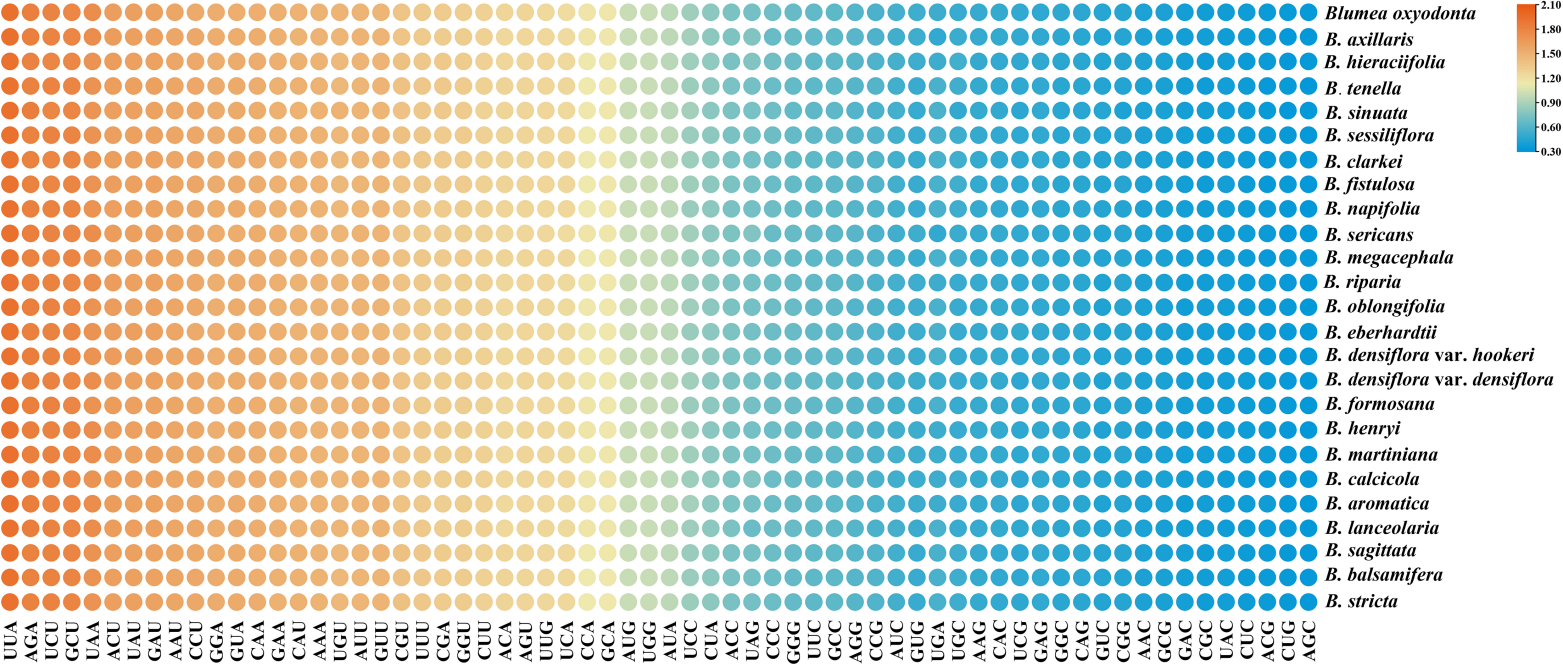
The RSCU values of codon in *Blumea* plastome. Color circle, the orange represent higher RSCU values while the blue indicate lower values.

Codon usage bias analysis was performed based on 79 shared protein-coding genes (PCGs) from 25 *Blumea* plastomes. Total length of the protein-coding sequences ranged from 67,977 to 68,031 bp, encoding 22,575–22,602 codons (**Supplementary Table 9**). Leucine (Leu) had the most codons (2,397–2,411), while cysteine (Cys) had the fewest (244–250). Methionine (Met) and tryptophan (Trp) were exclusively encoded by AUG and UGG, respectively. A total of 64 types of codons were identified across the 25 *Blumea* plastomes, with RSCU values ranging from 0.34 to 1.94 (**Figure 6**; **Supplementary Table 9**). Thirty codons (UUA, AGA, UCU, GCU, UAA, ACU, UAU, GAU, AAU, CCU, GGA, GUA, CAA, GAA, CAU, AAA, UGU, AUU, GUU, CGU, UUU, CGA, GGU, CUU, ACA, AGU, UUG, UCA, CCA, GCA) had RSCU > 1. Except for the codon UUG, all codons with RSCU > 1 preferred codons were A/U-ending. Furthermore, AUG and UGG exhibited an RSCU of 1, indicating no codon usage bias for these two codons.

### 3.5 Phylogenetic analyses

As shown in **Supplementary Table 10**, the plastome alignment matrix was 157,779 bp in length, containing 9,788 variable sites (5.97%) and 7,908 parsimony-informative sites (4.83%). The nrDNA alignment matrix was 4,171 bp in length, with 488 variable sites (8.31%) and 337 parsimony-informative sites (5.74%). Both ML and BI analyses based on the complete plastome and nrDNA yielded largely consistent phylogeny topologies, respectively, with the monophyly of *Blumea* strongly supported (BS = 100, PP = 1.00).

**Figure 7:**
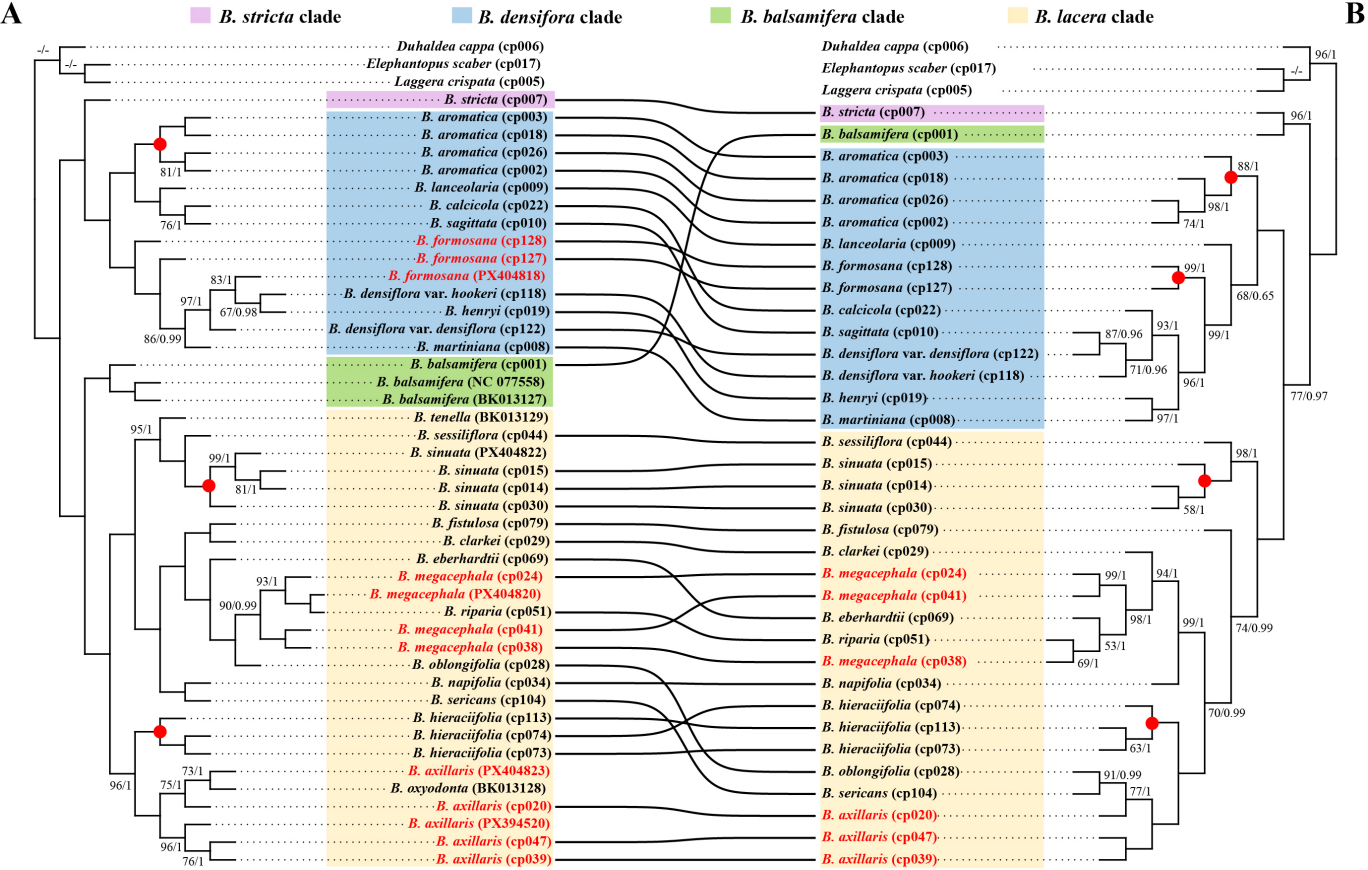
Phylogeny inferred from maximum likelihood (ML) and Bayesian (BI) analyses based on the complete plastomes **(A)** and nrDNA **(B)** of *Blumea*. The ML bootstrap values (BS) and the posterior probabilities values (PP) equal to 100/1 is omitted from each node and is not shown. “-/-” indicates that the value is missing. Species with highlighted in red represent the non-monophyletic species, while red circle on the node represent the monophyletic species. Samples with identical voucher numbers connected by curves.

In the plastome phylogenetic tree (**Figures 7A**), *Blumea* was divided into four clades: (1) the *B. stricta* clade, consisting of *B. stricta* (BS = 100, PP = 1.00); (2) the *B. densiflora* clade, sister to the *B. stricta* clade, comprising *B. aromatica*, *B. calcicola*, *B. densiflora* var. *densiflora*, *B. densiflora* var. *hookeri*, *B. formosana*, *B. henryi*, *B. lanceolaria*, *B. martiniana*, and *B. sagittata* (BS = 100, PP = 1.00). (3) the *B. balsamifera* clade containing *B. balsamifera* (BS = 100, PP = 1.00); (4) the *B. lacera* clade including *B. axillaris*, *B. clarkei*, *B. eberhardtii*, *B. fistulosa*, *B. hieraciifolia*, *B. megacephala*, *B. napifolia*, *B. oblongifolia*, *B. oxyodonta*, *B. riparia*, *B. sericans*, *B. sessiliflora*, *B. sinuata*, and *B. tenella* (BS = 100, PP = 1.00).

Within the *B. densiflora* clade, *B. lanceolaria* was sister to the *B. calcicola + B. sagittata* clade, and this combined clade then clustered with *B. aromatica.* The remaining species formed another clade, in which *B. formosana* (cp127), *B. formosana* (cp128), *B. martiniana*, and *B. densiflora* var. *densiflora*, *B. formosana* (PX404818) were successively resolved as sister lineages to the *B. henryi + B. densiflora* var. *hookeri* clade. In the *B. lacera* clade, *B. oxyodonta* was nested within the *B. axillaris* and clustered with *B. hieraciifolia*at the base of the *B. lacera* clade; *B. tenella and B. sessiliflora* were successively sister to *B. sinuata*, forming the next diverging clade, followed by the *B. napifolia* + *B. sericans* clade, the *B. fistulosa* + *B. clarkei* clade, *B. eberhardtii*, and *B. oblongifolia*; Finally, *B. riparia* was nested in *B. megacephala*, forming the terminal clade.

In the nrDNA-based phylogenetic tree, *Blumea* was likewise divided into the same four clades with the same taxa, but their phylogenetic positions were obviously different (**Figure 7B**). Specifically, the *B. stricta* clade was sister to the *B. balsamifera* clade (BS = 100, PP = 1.00), together constituting an early-diverging lineage. In contrast, the *B. densiflora* clade clustered with the *B. lacera* clade (BS = 100, PP = 1.00). Notably, substantial topological incongruence was observed between the cpDNA- and nrDNA-based trees within both the *B. densiflora* and the *B. lacera* clades. In the *B. densiflora* clade, *B. aromatica* formed the earliest-diverging lineage, followed by *B. lanceolaria*, *B. formosana*, and the *B. henryi* + *B. martiniana* clade; *B. calcicola* and *B. densiflora* var. *hookeri* were successively sister to the *B. sagittata* + *B. densiflora* var. *densiflora* clade. Within the *B. lacera* clade, *B. sessiliflora was* sister to *B. sinuata* at the base of the clade, followed by *B. fistulosa*; Among the remaining taxa, *B. napifolia and B. clarkei* were successively resolved as sister to a clade in which *B. eberhardtii* and *B. riparia* were nested in *B. megacephala*; Unlike in plastome-based phylogeny, *B. sericans* and *B. oblongifolia* were nested in *B. axillaris*, and together formed a clade sister *B. hieraciifolia.*

## 4 Discussion

### 4.1 Conservation in *Blumea* plastome

All *Blumea* plastomes exhibited a typical circular quadripartite structure, and were highly conserved in genome size, GC content and gene composition. Comparative junction analysis and Mauve analysis further confirmed the high structural conservation of *Blumea* plastomes, with no obvious contraction or expansion of IR regions, nor gene rearrangements detected. mVISTA analysis indicated high sequence similarity among genomes. In addition, the plastomes of this genus showed conservation in codon usage bias, characterized by identical preferred codon types, RSCU values, and the overall comparable distribution patterns and nucleotide repeat compositions of SSRs. These conserved features have also been reported in other plastomes of Asteraceae species (Thode et al., 2021; Duan et al., 2022; Yun and Kim, 2022; Xue et al., 2025).

### 4.2 Variations across *Blumea* plastome

Nevertheless, the plastomes of *Blumea* also displayed distinct, even unique, patterns of variation. Although the genes flanking each junction were identical, the junction regions across the genus could be divided into four types. Unexpectedly, these four types corresponded exactly to the four clades: Type I corresponded to the *B. lacera* clade; Type II corresponded to the *B. densiflora* clade; and Type III and Type IV to the independent *B. balsamifera* clade and *B. purpurea* clade, respectively, consistent with their distinct lineages in the phylogenetic tree. These results indicate that junction specificity provides valuable information for clade delimitation and offers useful supplementary evidence for phylogenetic inference. Similar patterns have been reported in other plant lineages (Thode and Lohmann, 2019; Liu et al., 2022; Xu et al., 2025).

Nucleotide diversity (Pi) analysis revealed the degree of variation in the nucleotide sequence. In this study, nine mutation hotspot regions (*trnH-psbA*, *ycf1*, *trnC-petN*, *trnY-rpoB*, *ycf2-trnL*, *ndhF-rpl32*, *rpl32-trnL*, *ccsA-ndhD*, and *ndhG-ndhI*) were identified, which can be employed as candidate DNA barcodes for species identification in *Blumea*. Among them, *trnH*-*psbA* represents a core universal DNA barcode widely used in previous studies (CBOL Plant Working Group, 2009; Hollingsworth et al., 2009; Thakur et al., 2019); the *ycf1* gene usually with the highest number of variable and parsimony-informative sites, was considered one of the most promising barcodes for species discrimination (Dong et al., 2015; Gogoi et al., 2020; Li et al., 2021); the *trnY-rpoB* region was less frequently mentioned, but the *trnY-trnE* and the *trnE*-*rpoB* regions within it had been recognized as mutation hotspots (Shen et al., 2020; Zheng et al., 2024), as had the other regions identified here (Jiao et al., 2018; Gao et al., 2020; Xie et al., 2021; Wei et al., 2022; Yao et al., 2022; Wang et al., 2024).

As one of the most widely used molecular markers, SSRs play important roles in species identification, population genetics, biodiversity research, and marker-assisted selection in molecular breeding (He et al., 2020; Li et al., 2022; Pan et al., 2024; Xu et al., 2024). In this study, extensive interspecific polymorphism of plastomic SSRs was detected among *Blumea* species. Firstly, substantial variation existed in both the total number of SSRs across plastomes and the number of SSR types, particularly mononucleotide repeats (**Supplementary Table 8**). Secondly, pentanucleotides repeats were scattered in some species of the *B. densiflora* clade and *B. lacera* clade, and hexanucleotide repeats were uniquely present in *B. henryi.* In addition, the SSR motifs were diverse, even specific across *Blumea* plastomes (**Supplementary Table 8**). Some unique SSR motifs were present in the plastomes of certain species—for instance, the TTTA AA motif in *B. henryi*, ATAAA/TATTT in *B. tenella*, and TTCT/ATCT in *B. fistulosa*—and may serve as species-specific molecular markers. Intriguingly, analogous to the junction types between the LSC, SSC, and IR regions, the distribution patterns of certain SSR motifs across *Blumea* plastomes appear to align with phylogenetic relationships. For instance, the G motif is exclusively detected in the *B. densiflor*a clade, while the TG motif was restricted to the *B. lacera* clade. Similarly, the ATTT and TATAT motifs also showed clade-specific distributions.

### 4.3 Phylogenetic implications

Compared with the previous fragment-based phylogeny of *Blumea*, the phylogenetic trees constructed from complete plastomes and nrDNA sequences in this study exhibited high node support and topological resolution. Our results also corroborated the taxonomic treatment of the *B. lacera*, *B. balsamifera*, and *B. densiflora* clades within the genus (Pornpongrungrueng et al., 2007; Pornpongrungrueng et al., 2009; Chung et al., 2022). Furthermore, both plastome-based and nrDNA-based trees supported the inclusion of *Cyathocline* Cass. within *Blumea* (Li et al., 2014), thus supporting the recognition of the *B. stricta* clade. Among these four clades, life form patterns were consistent with phylogenetic relationships. The *B. lacera* clade consisted of herbaceous or vine-like taxa; the *B. densiflora* clade comprised subshrubby species; and the *B. balsamifera* and *B. stricta* clades represented two independent evolutionary lineages corresponding to subshrubs/shrubs (*B. balsamifera*) and herbs (*B. stricta*), respectively.

Previously, the monophyly of taxa including *B. formosana*, *B. sinuata*, *B. megacephala*, *B. aromatica*, *B. lacera*, *B. axillaris*, *B. saxatili*s, and *B. hieraciifolia* was rarely well supported (Pornpongrungrueng et al., 2007; Pornpongrungrueng et al., 2009; Chung et al., 2022). In contrast, our plastome and nrDNA dataset greatly improved the resolution of this phylogenetic uncertainty. As expected, the monophyly of *B. sinuata*, *B. aromatica*, and *B. hieraciifolia* was strongly supported (BS = 100, PP = 1.00) in both trees, although *B. axillaris*, and *B. megacephala* still remained non-monophyletic. Notably, the *B. formosana*, which was non-monophyletic in the plastome tree, was resolved as monophyletic in the nrDNA tree, suggesting that nrDNA sequences may have greater power for resolving phylogenetic relationships in *Blumea*.

Nonetheless, the phenomenon of non-monophyly implied the limitations of using complete plastome or nrDNA in the genus. For recently diverged groups with complex evolutionary histories, such as *Panax* (Ji et al., 2019) and *Fargesia* (Lv et al., 2023), plastomes and nrDNA failed to clearly distinguish closely related species or robustly resolve phylogenetic relationships. Indeed, *Blumea* originated approximately 49.00–18.43 Ma, representing a relatively young genus that underwent rapid radiation during the Miocene (Zhang et al., 2019). Moreover, Chung et al. (2022) proposed that hybridization, introgression, and genetic exchange between widespread and endemic *Blumea* species may underlie paraphyly (such as *B. formosana* in plastome-based tree) in the genus. The cytonuclear discordance observed in our study (**Figure 7**) further supports the possible occurrence of hybridization, introgression, and incomplete lineage sorting (ILS) during the evolutionary history of *Blumea*.

## 5 Conclusion

This study presents the most comprehensive comparative analysis of plastomes from *Blumea* to date, systematically revealing the evolutionary characteristics of the plastomes within this genus, including their conservation and divergence. Phylogenetic reconstructions based on plastome and nrDNA datasets established a robust phylogenetic framework for *Blumea*, encompassing the *B. lacera* clade, *B. balsamifera* clade, *B. densiflora* clade, and *B. stricta* clade, and preliminarily clarified the phylogenetic relationships among species. Furthermore, the monophyly of taxa including *B. formosana*, *B. sinuata*, *B. megacephala*, *B. aromatica*, *B. axillaris*, and *B. hieraciifolia* was well examined. In summary, this study provides valuable genetic resources for *Blumea* and yields novel insights into its phylogeny, laying a solid foundation for subsequent taxonomic, systematic, and species identification research. To better elucidate the evolutionary history of *Blumea*, future studies with more sampling and integration of more genomic data, such as mitochondrial genome (mtDNA) or nuclear single-copy gene data, will be necessary.

## Data Availability

The data presented in the study are deposited in the Genbank, accession number PZ169104–PZ169136, PZ162812–PZ162849, PX776216–PX776217, and PX776219–PX776221.

## Supplementary material

Supplementary Figure 1 Mauve alignment of plastomes from 23 species and 2 varieties of *Blumea* species and outgroups. Rectangular blocks of the same color indicate collinear regions of sequences and the histograms within each block indicate the degree of sequence similarity.

Supplementary Table 1 Collecting information and GenBank accession numbers of the 38 samples of in this study.

Supplementary Table 2 Plastomes in phylogenetic analyses from GenBank.

Supplementary Table 3 Representative 25 plastomes of *Blumea* for comparative analyses.

Supplementary Table 4 Summary of Illumina sequencing for plastomes of 38 samples.

Supplementary Table 5 List of genes identified of plastomes in *Blumea*.

Supplementary Table 6 Features of nrDNA arrays in this study.

Supplementary Table 7 Features of junction sites of plastomes.

Supplementary Table 8 SSRs identified in *Blumea* plastomes.

Supplementary Table 9 Statistics of codon usage and RSCU of protein-coding regions of 25 *Blumea* plastomes.

Supplementary Table 10 Characteristics of the alignment matrix of complete plastome, and nrDNA involved in the phylogenetic analyses.
