# Supplementary figures and images for: Comparative plastome analyses and phylogenetic insights of Blumea DC
Source: Front Plant Sci. 2026 May 7;17:1835658. doi: 10.3389/fpls.2026.1835658 (PMC13190592; doi:10.3389/fpls.2026.1835658)

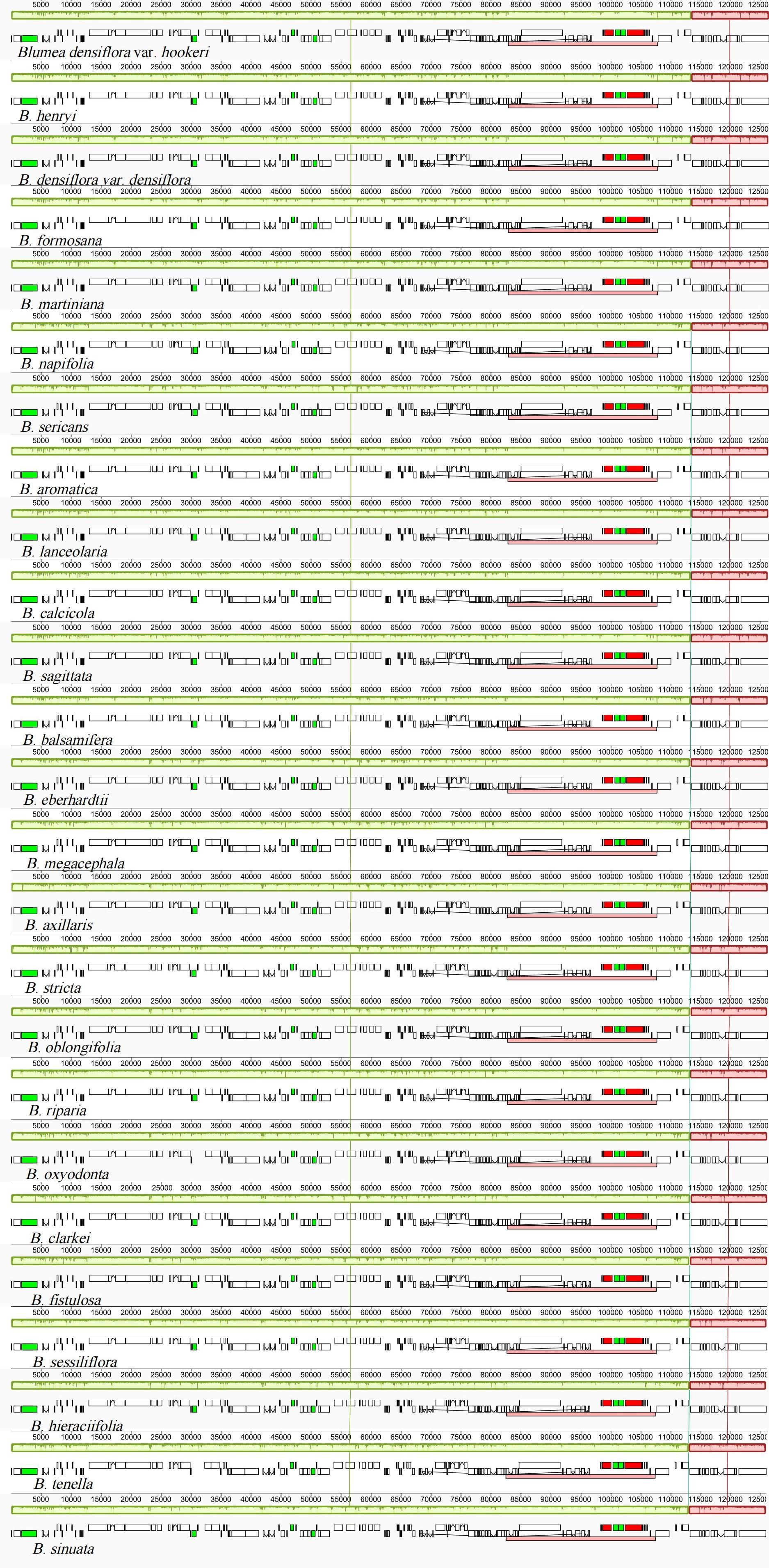

Supplement: Supplementary Figure 1 — Mauve alignment of plastomes from 23 species and 2 varieties of Blumea species and outgroups. Rectangular blocks of the same color indicate collinear regions of sequences and the histograms within each block indicate the degree of sequence similarity. [file Image1.jpeg]
