# Supplementary material for: Comparative plastome analyses and phylogenetic insights of Blumea DC
Source: Front Plant Sci. 2026 May 7;17:1835658. doi: 10.3389/fpls.2026.1835658 (PMC13190592; doi:10.3389/fpls.2026.1835658)
Supplement: Supplementary Table 1 — Collecting information and GenBank accession numbers of the 38 samples of in this study. [file Table1.docx]

**Supplementary Table 1 Collecting information and GenBank accession numbers of the 38 samples of in this study**

| **Species** | **Locality** | **Specimen Voucher** | **Plastome GenBank Accession** | **nrDNA GenBank Accession** |
| --- | --- | --- | --- | --- |
| *B. balsamifera* | Luodian,Guizhou | cp001 | PZ169104 | PZ162812 |
| *B. aromatica* | Guanling,Guizhou | cp002 | PZ169105 | PZ162813 |
| *B. aromatica* | Guanling,Guizhou | cp003 | PZ169106 | PZ162814 |
| *B. stricta* | Wangmo,Guizhou | cp007 | PZ169109 | PZ162817 |
| *B. martiniana* | Xing yi,Guizho | cp008 | PZ169110 | PZ162818 |
| *B. lanceolaria* | Wangmo,Guizhou | cp009 | PX776216 | PZ162819 |
| *B. sagittata* | Wangmo,Guizhou | cp010 | PZ169111 | PZ162820 |
| *B. sinuata* | Ceheng,Guizhou | cp014 | PX776217 | PZ162821 |
| *B. sinuata* | Wangmo,Guizhou | cp015 | PZ169112 | PZ162822 |
| *B. aromatica* | Luodian,Guizhou | cp018 | PZ169114 | PZ162824 |
| *B. henryi* | Luodian,Guizhou | cp019 | PZ169115 | PZ162825 |
| *B. axillaris* | Danzhou,Haina | cp020 | PZ169116 | PZ162826 |
| *B. calcicola* | Long'an,Guangxi | cp022 | PZ169117 | PZ162827 |
| *B. megacephala* | Guangzhou,Guangdong | cp024 | PZ169118 | PZ162828 |
| *B. aromatica* | Guangzhou,Guangdong | cp026 | PZ169119 | PZ162829 |
| *B. oblongifolia* | Guangzhou,Guangdong | cp028 | PX776219 | PZ162830 |
| *B. clarkei* | Guangzhou,Guangdong | cp029 | PX776220 | PZ162831 |
| *B. sinuata* | Mengla,Yunnan | cp030 | PZ169120 | PZ162832 |
| *B. napifolia* | Mengla,Yunnan | cp034 | PZ169121 | PZ162833 |
| *B. megacephala* | Nanning,Guangxi | cp038 | PZ169122 | PZ162834 |
| *B. axillaris* | Nanning,Guangxi | cp039 | PX776221 | PZ162835 |
| *B. megacephala* | Guangzhou,Guangdong | cp041 | PZ169123 | PZ162836 |
| *B. sessiliflora* | Guangzhou,Guangdong | cp044 | PZ169124 | PZ162837 |
| *B. axillaris* | Luodian,Guizhou | cp047 | PZ169125 | PZ162838 |
| *B. riparia* | Baise,Guangxi | cp051 | PZ169126 | PZ162839 |
| *B. eberhardtii* | Baise,Guangxi | cp069 | PZ169127 | PZ162840 |
| *B. hieraciifolia* | Funing,Yunnan | cp073 | PZ169128 | PZ162841 |
| *B. hieraciifolia* | Napo,Guangxi | cp074 | PZ169129 | PZ162842 |
| *B. fistulosa* | Funing,Yunnan | cp079 | PZ169130 | PZ162843 |
| *B. sericans* | Liuzhou,Guangxi | cp104 | PZ169131 | PZ162844 |
| *B. hieraciifolia* | Funing,Yunnan | cp113 | PZ169132 | PZ162845 |
| *B. densiflora* var. *hookeri* | Tengchong,Yunnan | cp118 | PZ169133 | PZ162846 |
| *B. densiflora* var. *densiflora* | Tengchong,Yunnan | cp122 | PZ169134 | PZ162847 |
| *B. formosana* | Guilin,Guangxi | cp127 | PZ169135 | PZ162848 |
| *B. formosana* | Guilin,Guangxi | cp128 | PZ169136 | PZ162849 |
| *Laggera crispata* | Guanling,Guizhou | cp005 | PZ169107 | PZ162815 |
| *Duhaldea cappa* | Wangmo,Guizhou | cp006 | PZ169108 | PZ162816 |
| *Elephantopus scaber* | Luodian,Guizhou | cp017 | PZ169113 | PZ162823 |
