# Supplementary material for: Comparative plastome analyses and phylogenetic insights of Blumea DC
Source: Front Plant Sci. 2026 May 7;17:1835658. doi: 10.3389/fpls.2026.1835658 (PMC13190592; doi:10.3389/fpls.2026.1835658)
Supplement: Supplementary Table 2 — Plastomes in phylogenetic analyses from GenBank. [file Table2.doc]

**Supplementary Table 2. Plastomes in phylogenetic analyses from GenBank**

| **Species** | **Family** | **GenBank accession** |
| --- | --- | --- |
| *B. axillaris* | *Asteraceae* | PX394520 |
| *B. axillaris* | Asteraceae | PX404823 |
| *B. balsamifera* | Asteraceae | NC_077558 |
| *B. formosana* | Asteraceae | PX404818 |
| *B. megacephala* | Asteraceae | PX404820 |
| *B. sinuata* | Asteraceae | PX404822 |
| *B. balsamifera* | Asteraceae | BK013127 |
| *B. oxyodonta* | Asteraceae | BK013128 |
| *B. tenella* | Asteraceae | BK013129 |
