# Supplementary material for: Comparative plastome analyses and phylogenetic insights of Blumea DC
Source: Front Plant Sci. 2026 May 7;17:1835658. doi: 10.3389/fpls.2026.1835658 (PMC13190592; doi:10.3389/fpls.2026.1835658)
Supplement: Supplementary Table 3 — Representative 25 plastomes of Blumea for comparative analyses. [file Table3.docx]

**Supplementary Table 3 Representative 25 plastomes of *Blumea* for comparative analyses**

| Species | Voucher/GenBank accession |
| --- | --- |
| *Blumea oxyodonta* | BK013128 |
| *Blumea tenella* | BK013129 |
| *Blumea balsamifera* | cp001 |
| *Blumea aromatica* | cp002 |
| *Blumea stricta* | cp007 |
| *Blumea martiniana* | cp008 |
| *Blumea lanceolaria* | cp009 |
| *Blumea sagittata* | cp010 |
| *Blumea henryi* | cp019 |
| *Blumea axillaris* | cp020 |
| *Blumea calcicola* | cp022 |
| *Blumea oblongifolia* | cp028 |
| *Blumea clarkei* | cp029 |
| *Blumea sinuata* | cp030 |
| *Blumea napifolia* | cp034 |
| *Blumea megacephala* | cp041 |
| *Blumea sessiliflora* | cp044 |
| *Blumea riparia* | cp051 |
| *Blumea eberhardtii* | cp069 |
| *Blumea hieraciifolia* | cp073 |
| *Blumea fistulosa* | cp079 |
| *Blumea sericans* | cp104 |
| *Blumea densiflora* var. *hookeri* | cp118 |
| *Blumea densiflora* var. *densiflora* | cp122 |
| *Blumea formosana* | cp127 |
