# Supplementary material for: Comparative plastome analyses and phylogenetic insights of Blumea DC
Source: Front Plant Sci. 2026 May 7;17:1835658. doi: 10.3389/fpls.2026.1835658 (PMC13190592; doi:10.3389/fpls.2026.1835658)
Supplement: Supplementary Table 4 — Summary of Illumina sequencing for plastomes of 38 samples. [file Table4.docx]

**Supplementary Table 4 Summary of Illumina sequencing for plastomes of 38 samples**

| **Samples** | **Species** | **No. of total of reads** | **No. of mapped reads** | **Size of chloroplast genome (bp)** | **Average coverage(×)** |
| --- | --- | --- | --- | --- | --- |
| cp001 | *B. balsamifera* | 32,388,020 | 2,036,297 | 151,171 | 2,033.994 |
| cp002 | *B. aromatica* | 40,938,902 | 2,583,049 | 151,186 | 2,579.871 |
| cp003 | *B. aromatica* | 29,537,944 | 909,591 | 151,186 | 908.472 |
| cp007 | *B. stricta* | 37,541,142 | 1,206,953 | 151,087 | 1,206.258 |
| cp008 | *B. martiniana* | 30,400,658 | 306,837 | 151,251 | 306.328 |
| cp009 | *B. lanceolaria* | 41,070,216 | 405,577 | 151,170 | 405.121 |
| cp010 | *B. sagittata* | 43,342,176 | 286,937 | 151,144 | 286.664 |
| cp014 | *B. sinuata* | 33,477,202 | 1,987,728 | 150,788 | 1,990.523 |
| cp015 | *B. sinuata* | 35,859,364 | 1,033,964 | 150,790 | 1,035.404 |
| cp018 | *B. aromatica* | 31,948,048 | 858,526 | 151,186 | 857.470 |
| cp019 | *B. henryi* | 30,394,400 | 135,225 | 151,269 | 134.985 |
| cp020 | *B. axillaris* | 32,887,514 | 1,512,477 | 151,054 | 1,511.936 |
| cp022 | *B. calcicola* | 35,154,108 | 429,367 | 151,169 | 428.887 |
| cp024 | *B. megacephala* | 31,854,086 | 152,124 | 151,077 | 152.046 |
| cp026 | *B. aromatica* | 33,452,172 | 237,891 | 151,186 | 237.598 |
| cp028 | *B. oblongifolia* | 32,123,396 | 287,159 | 151,067 | 287.032 |
| cp029 | *B. clarkei* | 33,736,044 | 186,815 | 151,017 | 186.794 |
| cp030 | *B. sinuata* | 34,942,162 | 1,024,402 | 150,779 | 1,025.903 |
| cp034 | *B. napifolia* | 27,587,306 | 1,360,696 | 151,232 | 1,358.609 |
| cp038 | *B. megacephala* | 29,136,876 | 1,240,547 | 151,071 | 1,239.964 |
| cp039 | *B. axillaris* | 30,819,090 | 1,471,990 | 151,043 | 1,471.571 |
| cp041 | *B. megacephala* | 29,068,572 | 1,587,717 | 151,079 | 1,586.887 |
| cp044 | *B. sessiliflora* | 28,862,604 | 729,210 | 150,925 | 729.572 |
| cp047 | *B. axillaris* | 29,136,436 | 3,469,546 | 151,049 | 3,468.420 |
| cp051 | *B. riparia* | 30,321,052 | 1,069,758 | 151,046 | 1,069.432 |
| cp069 | *B. eberhardtii* | 34,572,208 | 958,484 | 151,118 | 957.736 |
| cp073 | *B. hieraciifolia* | 34,651,680 | 993,749 | 150,874 | 994.579 |
| cp074 | *B. hieraciifolia* | 39,546,196 | 2,192,745 | 150,871 | 2,194.620 |
| cp079 | *B. fistulosa* | 65,316,804 | 955,262 | 150,992 | 955.313 |
| cp104 | *B. sericans* | 29,361,066 | 493,163 | 151,211 | 492.475 |
| cp113 | *B. hieraciifolia* | 30,042,010 | 558,810 | 150,865 | 559.310 |
| cp118 | *B. densiflora* var. hookeri | 30,128,422 | 289,372 | 151,272 | 288.852 |
| cp122 | *B. densiflora* var. densiflora | 30,296,690 | 142,896 | 151,264 | 142.647 |
| cp127 | *B. formosana* | 30,445,780 | 202,025 | 151,260 | 201.678 |
| cp128 | *B. formosana* | 30,466,442 | 250,311 | 151,256 | 249.887 |
| cp005 | *Laggera crispata* | 30,950,910 | 1,518,364 | 152,433 | 1,504.09 |
| cp006 | *Duhaldea cappa* | 34,305,498 | 133,701 | 150,864 | 133.822 |
| cp017 | *Elephantopus scaber* | 31,903,326 | 272,025 | 151,910 | 270.395 |
