# Supplementary material for: Comparative plastome analyses and phylogenetic insights of Blumea DC
Source: Front Plant Sci. 2026 May 7;17:1835658. doi: 10.3389/fpls.2026.1835658 (PMC13190592; doi:10.3389/fpls.2026.1835658)
Supplement: Supplementary Table 5 — List of genes identified of plastomes in Blumea. [file Table5.docx]

**Supplementary Table 5 List of genes identified of plastomes in *Blumea***

| **Genes category** | **Gene group** | **Genes** |
| --- | --- | --- |
| Self-replication | Ribosomal RNA genes | *rrn*4.5×2*,* 5×2*,* 16×2*,* 23×2 |
|  | Transfer RNA genes | *trn*A-UGC* ×2*, trn*C-GCA*, trn*D-GUC*, trn*E-UUC*, trn*F-GAA*, trn*fM-CAU*, trn*G-GCC*, trn*G-UCC**, trn*H-GUG*, trn*I-CAU*×*2*, trn*I-GAU* ×2*, trn*K-UUU**, trn*L-CAA×2*,* *trn*L-UAA**, trn*L-UAG*, trn*M-CAU*, trn*N-GUU×2*, trn*P-UGG*, trn*Q-UUG*, trn*R-ACG×2*, trn*R-UCU*, trn*S-GCU*, trn*S-GGA*, trn*S-UGA*, trn*T-UGU*, trn*T-GGU*, trn*V-GAC×2*, trn*V-UAC**, trn*W-CCA*, trn*Y-GUA |
|  | Ribosomal protein (small subunit) | *rps*2*,* 3*,* 4*,* 7×2*,* 8*,* 11*,* 12** ×2*,* 14*,* 15*,* 16**,* 18*,* 19 |
|  | Ribosomal protein (large subunit) | *rpl*2*×2*,* 14*,* 16**,* 20*,* 22*,* 23×2*,* 32*,* 33*,* 36 |
|  | RNA polymerase | *rpo*A*,* B*,* C1**,* C2 |
|  | Translational initiation factor | *inf*A |
| Genes for photosynthesis | Subunits of photosystem I | *psa*A*,* B*,* C*,* I*，*J*, ycf*3***, ycf*4 |
|  | Subunits of photosystem II | *psb*A*,* B*,* C*,* D*,* E*,* F*,* H*,* I*,* J*,* K*,* L*,* M*,* N*,* T*,* Z |
|  | Subunits of cytochrome | *pet*A*,* B**,* D**,* G*,* L*,* N |
|  | Subunits of ATP synthase | *atp*A*,* B*,* E*,* F**,* H*,* I |
|  | Large subunit of Rubisco | *rbc*L |
|  | Subunits of NADH dehydrogenase | *ndh*A**,* B* ×2*,* C*,* D*,* E*,* F*,* G*,* H*,* I*,* J*,* K |
| Other genes | Maturase | *mat*K |
|  | Envelope membrane protein | *cem*A |
|  | Subunit of acetyl-CoA | *acc*D |
|  | Synthesis gene | *ccs*A |
|  | ATP-dependent protease | *clp*P**** |
|  | Component of TIC complex | *ycf*1^a^ |
|  | Component of 2-MD heteromeric AAA-ATPase complex | *ycf*2×2 |

Notes: ×2: Two gene copies in IR regions; *: With one intron; **: With two introns; ^a^: partially duplicated genes; ^ψ^: Pseudogene
