# Supplementary material for: Comparative plastome analyses and phylogenetic insights of Blumea DC
Source: Front Plant Sci. 2026 May 7;17:1835658. doi: 10.3389/fpls.2026.1835658 (PMC13190592; doi:10.3389/fpls.2026.1835658)
Supplement: Supplementary Table 6 — Features of nrDNA arrays in this study. [file Table6.docx]

**Supplementary Table 6 Features of nrDNA arrays in this study**

| **Species** | **Isolate** | **Total** | |
| --- | --- | --- | --- |
|  |  | **Length (bp)** | **GC (%)** |
| *B. balsamifera* | cp001 | 5,856 | 53.5% |
| *B. aromatica* | cp002 | 5,851 | 53.3% |
| *B. aromatica* | cp003 | 5,851 | 53.3% |
| *B. purpurea* | cp007 | 5,848 | 53.0% |
| *B. martiniana* | cp008 | 5,851 | 53.2% |
| *B. lanceolaria* | cp009 | 5,851 | 53.3% |
| *B. sagittata* | cp010 | 5,851 | 53.2% |
| *B. sinuata* | cp014 | 5,851 | 52.6% |
| *B. sinuata* | cp015 | 5,851 | 52.6% |
| *B. aromatica* | cp018 | 5,851 | 53.3% |
| *B. henryi* | cp019 | 5,851 | 53.2% |
| *B. axillaris* | cp020 | 5,847 | 52.9% |
| *B. calcicola* | cp022 | 5,851 | 53.2% |
| *B. megacephala* | cp024 | 5,848 | 53.1% |
| *B. aromatica* | cp026 | 5,851 | 53.3% |
| *B. oblongifolia* | cp028 | 5,847 | 53.0% |
| *B. clarkei* | cp029 | 5,849 | 53.1% |
| *B. sinuata* | cp030 | 5,851 | 52.6% |
| *B. napifolia* | cp034 | 5,850 | 53.2% |
| *B. megacephala* | cp038 | 5,848 | 53.1% |
| *B. axillaris* | cp039 | 5,847 | 52.9% |
| *B. megacephala* | cp041 | 5,848 | 53.1% |
| *B. sessiliflora* | cp044 | 5,851 | 53.1% |
| *B. axillaris* | cp047 | 5,847 | 52.9% |
| *B. riparia* | cp051 | 5,848 | 53.1% |
| *B. eberhardtii* | cp069 | 5,846 | 53.1% |
| *B. hieraciifolia* | cp073 | 5,848 | 52.7% |
| *B. hieraciifolia* | cp074 | 5,848 | 52.7% |
| *B. fistulosa* | cp079 | 5,849 | 52.9% |
| *B. sericans* | cp104 | 5,847 | 53.0% |
| *B. hieraciifolia* | cp113 | 5,848 | 52.7% |
| *B. densiflora* var. *hookeri* | cp118 | 5,851 | 53.2% |
| *B. densiflora* var. *densiflora* | cp122 | 5,851 | 53.2% |
| *B. formosana* | cp127 | 5,851 | 53.2% |
| *B. formosana* | cp128 | 5,851 | 53.2% |
| *Laggera crispata* | cp005 | 5,849 | 53.9% |
| *Duhaldea cappa* | cp006 | 5,853 | 53.2% |
| *Elephantopus scaber* | cp017 | 5,844 | 53.9% |
