# Supplementary material for: Comparative plastome analyses and phylogenetic insights of Blumea DC
Source: Front Plant Sci. 2026 May 7;17:1835658. doi: 10.3389/fpls.2026.1835658 (PMC13190592; doi:10.3389/fpls.2026.1835658)
Supplement: Supplementary Table 7 — Features of junction sites of plastomes. [file Table7.docx]

**Supplementary Table 7 Features of junction sites of plastomes**

| Type | Voucher/accession number | Species | LSC-IRb | IRb-SSC | SSC-IRa | IRa-LSC |
| --- | --- | --- | --- | --- | --- | --- |
| Type Ⅰ | PX394520 | *B. axillaris* | *rps19* (10 bp); *rpl2* (54 bp) | *ycf1*; *ndhF* (2 bp) | *ycf1* (573 bp) | *rpl2* (54 bp); *trnH* (4 bp) |
|  | PX404823 | *B. axillaris* | *rps19* (10 bp); *rpl2* (54 bp) | *ycf1*; *ndhF* (2 bp) | *ycf1* (573 bp) | *rpl2* (54 bp); *trnH* (4 bp) |
|  | cp039 | *B. axillaris* | *rps19* (10 bp); *rpl2* (54 bp) | *ycf1*; *ndhF* (2 bp) | *ycf1* (573 bp) | *rpl2* (54 bp); *trnH* (4 bp) |
|  | cp047 | *B. axillaris* | *rps19* (10 bp); *rpl2* (54 bp) | *ycf1*; *ndhF* (2 bp) | *ycf1* (573 bp) | *rpl2* (54 bp); *trnH* (4 bp) |
|  | cp020 | *B. axillaris* | *rps19* (10 bp); *rpl2* (55 bp) | *ycf1*; *ndhF* (2 bp) | *ycf1* (573 bp) | *rpl2* (55 bp); *trnH* (4 bp) |
|  | cp029 | *B. clarkei* | *rps19* (10 bp); *rpl2* (54 bp) | *ycf1*; *ndhF* (2 bp) | *ycf1* (573 bp) | *rpl2* (54 bp); *trnH* (4 bp) |
|  | cp069 | *B. eberhardtii* | *rps19* (10 bp); *rpl2* (53 bp) | *ycf1*; *ndhF* (2 bp) | *ycf1* (573 bp) | *rpl2* (53 bp); *trnH* (4 bp) |
|  | cp079 | *B. fistulosa* | *rps19* (10 bp); *rpl2* (52 bp) | *ycf1*; *ndhF* (2 bp) | *ycf1* (573 bp) | *rpl2* (52 bp); *trnH* (4 bp) |
|  | cp073 | *B. hieraciifolia* | *rps19* (10 bp); *rpl2* (54 bp) | *ycf1*; *ndhF* (2 bp) | *ycf1* (573 bp) | *rpl2* (54 bp); *trnH* (4 bp) |
|  | cp074 | *B. hieraciifolia* | *rps19* (10 bp); *rpl2* (54 bp) | *ycf1*; *ndhF* (2 bp) | *ycf1* (573 bp) | *rpl2* (54 bp); *trnH* (4 bp) |
|  | cp113 | *B. hieraciifolia* | *rps19* (10 bp); *rpl2* (54 bp) | *ycf1*; *ndhF* (2 bp) | *ycf1* (573 bp) | *rpl2* (54 bp); *trnH* (4 bp) |
|  | PX404820 | *B. megacephala* | *rps19* (10 bp); *rpl2* (52 bp) | *ycf1*; *ndhF* (2 bp) | *ycf1* (573 bp) | *rpl2* (52 bp); *trnH* (4 bp) |
|  | cp024 | *B. megacephala* | *rps19* (10 bp); *rpl2* (52 bp) | *ycf1*; *ndhF* (2 bp) | *ycf1* (573 bp) | *rpl2* (52 bp); *trnH* (4 bp) |
|  | cp038 | *B. megacephala* | *rps19* (10 bp); *rpl2* (53 bp) | *ycf1*; *ndhF* (2 bp) | *ycf1* (573 bp) | *rpl2* (53 bp); *trnH* (4 bp) |
|  | cp041 | *B. megacephala* | *rps19* (10 bp); *rpl2* (53 bp) | *ycf1*; *ndhF* (2 bp) | *ycf1* (573 bp) | *rpl2* (53 bp); *trnH* (4 bp) |
|  | cp034 | *B. napifolia* | *rps19* (10 bp); *rpl2* (53 bp) | *ycf1*; *ndhF* (2 bp) | *ycf1* (573 bp) | *rpl2* (53 bp); *trnH* (4 bp) |
|  | cp028 | *B. oblongifolia* | *rps19* (10 bp); *rpl2* (52 bp) | *ycf1*; *ndhF* (2 bp) | *ycf1* (573 bp) | *rpl2* (52 bp); *trnH* (4 bp) |
|  | BK013128 | *B. oxyodonta* | *rps19* (10 bp); *rpl2* (54 bp) | *ycf1*; *ndhF* (2 bp) | *ycf1* (573 bp) | *rpl2* (54 bp); *trnH* (3 bp) |
|  | cp051 | *B. riparia* | *rps19* (10 bp); *rpl2* (52 bp) | *ycf1*; *ndhF* (2 bp) | *ycf1* (573 bp) | *rpl*2 (52 bp); *trnH* (4 bp) |
|  | cp104 | *B. sericans* | *rps19* (10 bp); *rpl2* (53 bp) | *ycf1*; *ndhF* (2 bp) | *ycf1* (573 bp) | *rpl2* (53 bp); *trnH* (4 bp) |
|  | cp044 | *B. sessiliflora* | *rps19* (10 bp); *rpl2* (55 bp) | *ycf1*; *ndhF* (1 bp) | *ycf1* (574 bp) | *rpl2* (55 bp); *trnH* (4 bp) |
|  | cp014 | *B. sinuata* | *rps19* (10 bp); *rpl2* (53 bp) | *ycf1*; *ndhF* (1 bp) | *ycf1* (574 bp) | *rpl2* (53 bp); *trnH* (4 bp) |
|  | cp015 | *B. sinuata* | *rps19* (10 bp); *rpl2* (53 bp) | *ycf1*; *ndhF* (1 bp) | *ycf1* (574 bp) | *rpl2* (53 bp); *trnH* (4 bp) |
|  | cp030 | *B. sinuata* | *rps19* (10 bp); *rpl2* (53 bp) | *ycf1*; *ndhF* (1 bp) | *ycf1* (574 bp) | *rpl2* (53 bp); *trnH* (4 bp) |
|  | PX404822 | *B. sinuata* | *rps19* (10 bp); *rpl2* (55 bp) | *ycf1*; *ndhF* (1 bp) | *ycf1* (574 bp) | *rpl2* (53 bp); *trnH* (4 bp) |
|  | BK013129 | *B. tenella* | *rps19* (10 bp); *rpl2* (55 bp) | *ycf1*; *ndhF* (2 bp) | *ycf1* (573 bp) | *rpl2* (55 bp); *trnH* (3 bp) |
| Type Ⅱ | cp002 | *B. aromatica* | *rps19* (13 bp); *rpl2* (54 bp) | *ycf1*; *ndhF* (29 bp) | *ycf1* (564 bp) | *rpl2* (54 bp); *trnH* (2 bp) |
|  | cp003 | *B. aromatica* | *rps19* (13 bp); *rpl2* (54 bp) | *ycf1*; *ndhF* (29 bp) | *ycf1* (564 bp) | *rpl2* (54 bp); *trnH* (2 bp) |
|  | cp018 | *B. aromatica* | *rps19* (13 bp); *rpl2* (54 bp) | *ycf1*; *ndhF* (29 bp) | *ycf1* (564 bp) | *rpl2* (54 bp); *trnH* (2 bp) |
|  | cp026 | *B. aromatica* | *rps19* (13 bp); *rpl2* (54 bp) | *ycf1*; *ndhF* (29 bp) | *ycf1* (564 bp) | *rpl2* (54 bp); *trnH* (2 bp) |
|  | cp022 | *B. calcicola* | *rps19* (13 bp); *rpl2* (55 bp) | *ycf1*; *ndhF* (40bp) | *ycf1* (564 bp) | *rpl2* (55 bp); *trnH* (2 bp) |
|  | cp122 | *B. densiflora* var. *densiflora* | *rps19* (13 bp); *rpl2* (53 bp) | *ycf1*; *ndhF* (40bp) | *ycf1* (564 bp) | *rpl2* (53 bp); *trnH* (2 bp) |
|  | cp118 | *B. densiflora* var. *hookeri* | *rps19* (13 bp); *rpl2* (53 bp) | *ycf1*; *ndhF* (40bp) | *ycf1* (564 bp) | *rpl2* (53 bp); *trnH* (2 bp) |
|  | cp127 | *B. formosana* | *rps19* (13 bp); *rpl2* (53 bp) | *ycf1*; *ndhF* (40bp) | *ycf1* (564 bp) | *rpl2* (53 bp); *trnH* (2 bp) |
|  | cp128 | *B. formosana* | *rps19* (13 bp); *rpl2* (53 bp) | *ycf1*; *ndhF* (40bp) | *ycf1* (564 bp) | *rpl2* (53 bp); *trnH* (2 bp) |
|  | PX404818 | *B. formosana* | *rps19* (13 bp); *rpl2* (53 bp) | *ycf1*; *ndhF* (40bp) | *ycf1* (564 bp) | *rpl2* (53 bp); *trnH* (2 bp) |
|  | cp019 | *B. henryi* | *rps19* (13 bp); *rpl2* (53 bp) | *ycf1*; *ndhF* (37bp) | *ycf1* (564 bp) | *rpl2* (53 bp); *trnH* (2 bp) |
|  | cp009 | *B. lanceolaria* | *rps19* (25 bp); *rpl2* (44 bp) | *ycf1*; *ndhF* (40bp) | *ycf1* (564 bp) | *rpl2* (44 bp); *trnH* (13 bp) |
|  | cp008 | *B. martiniana* | *rps19* (13 bp); *rpl2* (53 bp) | *ycf1*; *ndhF* (29 bp) | *ycf1* (564 bp) | *rpl2* (53 bp); *trnH* (2 bp) |
|  | cp010 | *B. sagittata* | *rps19* (13 bp); *rpl2* (55 bp) | *ycf1*; *ndhF* (29 bp) | *ycf1* (564 bp) | *rpl2* (53 bp); *trnH* (2 bp) |
| Type Ⅲ | BK013127 | *B. balsamifera* | *rps19* (11 bp); *rpl2* (60 bp) | *ycf1*; *ndhF* (16 bp) | *ycf1* (581 bp) | *rpl2* (60 bp); *trnH* (2 bp) |
|  | NC_077558 | *B. balsamifera* | *rps19* (11 bp); *rpl2* (60 bp) | *ycf1*; *ndhF* (16 bp) | *ycf1* (581 bp) | *rpl2* (60 bp); *trnH* (3 bp) |
|  | cp001 | *B. balsamifera* | *rps19* (11 bp); *rpl2* (61 bp) | *ycf1*; *ndhF* (16 bp) | *ycf1* (581 bp) | *rpl2* (61 bp); *trnH* (3 bp) |
| Type Ⅳ | cp007 | *B. stricta* | *rps19* (13 bp); *rpl2* (61 bp) | *ycf1; ndhF* (3 bp) | *ycf1* (574 bp) | *rpl2* (61 bp); *trnH* (1 bp) |
