# Supplementary material for: Comparative plastome analyses and phylogenetic insights of Blumea DC
Source: Front Plant Sci. 2026 May 7;17:1835658. doi: 10.3389/fpls.2026.1835658 (PMC13190592; doi:10.3389/fpls.2026.1835658)
Supplement: Supplementary Table 10 — Characteristics of the alignment matrix of complete plastome, and nrDNA involved in the phylogenetic analyses. [file Table10.docx]

**Supplementary Table 10 Characteristics of the alignment matrix of complete plastome, and nrDNA.**

| Sequence | Aligned length (bp) | No. of variable sites (divergence %) | No. of parsimony informative sites (divergence %) |
| --- | --- | --- | --- |
| Plastome | 157,779 | 9,823 | 5,088 |
| nrDNA | 4,171 | 546 | 394 |
